# 90 Years of progesterone: Ninety years of progesterone: the ‘other’ ovarian hormone

**DOI:** 10.1530/JME-20-0145

**Published:** 2020-06-01

**Authors:** Simak Ali, Kirsty Balachandran, Bert O’Malley

**Affiliations:** 1 Department of Surgery & Cancer, Imperial College London, London, UK; 2 Department of Molecular and Cellular Biology, Baylor College of Medicine, Houston, TX, USA

The beginning of the twentieth century saw a transformation in the field of endocrinology. Prominent among the many seminal discoveries was the identification and subsequent purification of ovarian steroid hormones, estrogen and progesterone. It is wholly 90 years since the first report of the isolation and characterisation of progesterone by Willard M. Allen and George W. Corner (Corner & Allen 1929). We celebrate this landmark discovery with a special issue of the *Journal of Molecular Endocrinology*, with reviews from international leaders in the field of endocrinology and progesterone receptor function. These reviews describe our current understanding of the molecular mechanisms by which progesterone exerts its cellular effects and the biological perspective for its actions in normal physiology and in disease. The reviews in this series sum up the advances made in understanding the physiological actions of progesterone and future research directions for greater understanding of this important hormone and attendant implications for human health.

The importance of progesterone in the female reproductive system and mammary gland development is now well-established, as contextualised in reviews by DeMayo and Lydon and by Brisken and Scabia (Brisken & Scabia 2020, DeMayo & Lydon 2020). Clinically, this understanding has led to the development of progesterone receptor modulating drugs with corresponding indications including contraception, termination of pregnancy, dysfunctional uterine bleeding and endometrial and breast cancers. Along the way, the use of synthetic progestins has led to controversy, most particularly its inclusion in hormone-replacement therapy (HRT) to prevent the potentially malignant effects of unopposed estrogen on the endometrium. Hence, the Women’s Health Initiative trial was terminated early when combined estrogen and medroxyprogesterone acetate was associated with an increased incidence of breast cancers, a greater percentage of patients with abnormal mammogram findings and a higher proportion of advanced stage breast cancers at diagnosis (Chlebowski *et al.* 2003). The results of the Million Women Study, published in the same year, revealed similarly concerning findings associating progestin-containing HRT with an increased incidence and mortality from breast cancer (Beral 2003).

The progesterone story begins with Willard Allen’s somewhat unorthodox entry into the University of Rochester medical school in 1926. Walking into the Dean’s office mid-term, without an appointment or application, Allen managed to persuade the Dean (George Whipple, the famed pathologist who would become a Nobel laureate 8 years later) to arrange an interview and secured a place for himself. On the same day Allen decided to try his luck with the Dean, he met George Corner, Professor of anatomy. With Allen being ‘both young and visionary’ (Allen 1974) and Corner the more experienced researcher, this chance encounter sparked a successful working relationship which really flourished when Allen accepted an anatomy fellowship later in his first year.

Five years after the description of the first major ovarian hormone, estrogen, in 1923, Allen and Corner’s first paper reported the ‘pro-gestational’ proliferative effect of corpus luteum extract on the endometria of castrated mature female rabbits, which was not seen with estrogen-rich follicular fluid or placental extracts (Corner & Allen 1929). Coincidentally, estrogen and progesterone were discovered by two Allens (Edgar Allen identified the former along with Edward Doisy). As Willard Allen was to go on to state ‘The two ALLENS are of separate lineage; the two female sex hormones are closely related’ (Allen 1974). After demonstrating that a component of the corpus luteum extract was responsible for maintaining the implantation and protection of blastocysts (Allen & Corner 1929), Willard Allen and Corner set about trying to isolate and purify the substance.

Through an arduous process of high-vacuum distillation and fractional crystallisation, Allen finally succeeded in isolating the newly identified hormone. However, Allen and Corner were no longer the sole team working on this; four international teams announced the isolation of the hormone in 1934 (Butenandt & Westphal 1934, Hartmann & Wettstein 1934, Slotta *et al.* 1934, Wintersteiner & Allen 1934) and shortly after the chemical formula was also deciphered. At a special meeting of the Health Organization of the League of Nations in 1935, the ovarian hormone that Allen and Corner had initially called ‘Progestin’ was formally named as ‘Progesterone’.

The structures of the steroid hormones were resolved through the 1930s, with each shown to be derived from cholesterol and therefore sharing highly similar structures (Miller & Auchus 2011). From this followed the delineation of the steroidogenesis pathway and the discovery that progesterone is not just a product of this process but also a critical upstream intermediate, essential for the biosynthesis of aldosterone, cortisol, estradiol and testosterone. Over the years, steroidogenic enzymes and regulatory proteins have been identified and further annotated our knowledge of this pathway. The inextricable association between progesterone synthesis and that of downstream mineralocorticoids, glucocorticoids, estrogens and androgens adds increased complexity to our understanding of the actions and interactions of these hormones.

The next major advance in the field came just over three decades later, with evidence that progesterone binds to a protein receptor, PR, reported by Bert O’Malley, Merry Sherman and David Toft using the chick oviduct model (O’Malley *et al.* 1970). Two isoforms were found once PR was purified, and to this day, their divergent functions have not been fully determined. Having contributed a large body of work to this field, Bert O’Malley recalls how these discoveries led to several landmark studies elucidating the mechanism of action of PR, the estrogen receptor (ER), the glucocorticoid receptor (GR) and the other nuclear receptors (NRs) (O’Malley 2020). O’Malley further describes the insights that revealed the regulation of PR expression by ER and the pivotal role of co-regulators in transcriptional regulation by NRs. The promise of PR and other NRs as potential therapeutic targets was beginning to be realised.

Historically, there has been confusion around classification of progesterone and the myriad other PR ligands. The terms progesterone, progestogens and progestins have sometimes, incorrectly, been used interchangeably. It is now broadly accepted that progesterone is the only native ligand, progestogens comprise all substances that activate PR and result in a progesterone-like effect and progestins are synthetic PR agonists (Carroll *et al.* 2017). After the development of Norethisterone in the early 1950s (Djerassi *et al.* 1954), a flurry of other potent progestins were developed, primarily for contraception, in the 1960s. SPRMs (selective progesterone receptor modulators) target PR in either an agonistic, antagonistic or mixed fashion. Critchley and Chodankar comprehensively review the development of key SPRMs and their clinical utility (Critchley & Chondankar 2020).

Despite progesterone being such a familiar name and ubiquitous in clinical practice, many questions remain unanswered, including its role in normal breast and gynaecological physiology. As its name suggests, progesterone is required for the maintenance of gestation but also, critically, for ensuring success of the earliest stage of pregnancy, blastocyst implantation. Understanding progesterone regulation of endometrial ‘receptivity’ and decidualization is imperative in investigating the aetiology of early miscarriage. DeMayo and Lydon review this fascinating process in detail and outline the key players involved in mediating uterine PR action (DeMayo & Lydon 2020). Similarly, questions remain about PR signaling pathways in the normal breast, an organ with unique plasticity given its ability to adapt through puberty, menstruation, pregnancy and lactation. These evolving hormonal environments create significant challenges for studying the effects of PR signaling in the breast and Cathrin Brisken’s team have pioneered several techniques and models to overcome these. She reviews these developments together with an overview on recognised mediators of PR signaling in normal breast physiology (Brisken & Scabia 2020).

As major contributors to the field over many years, Horwitz and Sartorius discuss why the role of progesterone in breast cancer remains controversial, with context-dependent observations (varying with experimental model, hormonal milieu, class and dose of PR ligand used) on its proliferative/anti-proliferative potential (Horwitz & Sartorius 2020). As the first prognostic and predictive marker of response to endocrine therapy for ER positive breast cancer, the importance of PR is now recognised not only as a marker of a functional ER, but also as a potential moderator of ER activity (Mohammed *et al.* 2015). The differentiation seen in normal breast tissue is thought to result from progesterone-regulated mammary stem cell populations. Horwitz and Sartorius review the evidence associating progesterone with expansion of breast cancer stem cells, a population strongly implicated in metastatic disease and tumour heterogeneity.

Given the ongoing uncertainty regarding the use of PR-modulating drugs in the treatment of breast cancer, the specific mechanisms through which PR mediates the effects of progesterone in breast cancer are of great interest. Beato *et al*. describe their important work in elucidating progesterone-directed chromatin remodelling, the advances in models used to investigate this to date and promising future approaches (Beato *et al.* 2020). Alongside their classical role as transcription factors, alternative non-genomic modes of action have been ascribed to steroid hormone receptors. Carol Lange and colleagues review how crosstalk with protein kinase directed signaling pathways impact on steroid receptor activities, through their detailed study of the effects of MAPK and CDK mediated PR phosphorylation and downstream cascades (Dwyer *et al*. 2020). The similarities between PR and GR are striking, with both NRs able to instigate ligand-independent, self-perpetuating signaling loops with the activation of downstream effectors inducing further phosphorylation of the original NR. Given the increase in MAPK and CDK signaling seen in breast cancer, this reveals another tantalising therapeutic strategy for breast cancer.

Discovery of progesterone 90 years ago has yielded remarkable insights into the physiological importance of this hormone and defined the molecular mechanisms by which progesterone actions are mediated. This research has had profound implications for human health, but as the reviews acknowledge, much remains to be revealed. The coming years promise further exciting insights into the ‘second’ ovarian hormone.

## Declaration of interest

The authors declare that there is no conflict of interest that could be perceived as prejudicing the impartiality of this editorial review.

## Funding

This research did not receive any specific grant from any funding agency in the public, commercial or not-for-profit sector.
